# A qualitative approach to understand antiretroviral therapy (ART) adherence for refugees living in Nakivale Refugee Settlement in Uganda

**DOI:** 10.1186/s13031-018-0145-1

**Published:** 2018-03-12

**Authors:** Kelli N. O’Laughlin, Shada A. Rouhani, Julius Kasozi, Kelsy E. Greenwald, Nicholas R. Perkons, Zikama M. Faustin, Ingrid V. Bassett, Norma C. Ware

**Affiliations:** 1 Division of International Emergency Medicine and Humanitarian Programs, Department of Emergency Medicine, Brigham and Women’s Hospital, 75 Francis Street, Boston, MA 02115 USA; 2 Medical Practice Evaluation Center, Department of Internal Medicine, Massachusetts General Hospital, 100 Cambridge Street, Boston, MA 02114 USA; 3 Harvard Medical School, 25 Shattuck Street, Boston, MA 02115 USA; 4 United Nations High Commissioner for Refugees, P.O. Box 3813, Kampala, Uganda; 5 Harvard University, Massachusetts Hall, Boston, MA 02138 USA; 6 Bugema University, P.O. Box 6529, Kampala, Uganda; 7 Division of Infectious Disease, Department of Medicine, Massachusetts General Hospital, 55 Fruit Street, Boston, MA 02114 USA; 8 Harvard University Center for AIDS Research, 42 Church Street, Cambridge, MA 0213 USA; 9 Department of Medicine, Brigham and Women’s Hospital, 75 Francis Street, Boston, MA 02115 USA; 10 Department of Global Health & Social Medicine, Harvard Medical School, 25 Shattuck Street, Boston, MA 02115 USA; 11 Harvard Affiliated Emergency Medicine Residency, 75 Francis Street, Boston, MA 02115 USA

**Keywords:** HIV, AIDS, Refugee, Migration, Antiretroviral therapy, ART, ART adherence, Africa, Uganda, Sub-Saharan Africa

## Abstract

**Background:**

Refugees living with HIV in sub-Saharan Africa suffer unique hardships that may increase their vulnerability to interruptions in antiretroviral therapy (ART).

**Methods:**

To investigate refugees’ experiences adhering to ART, we conducted inperson interviews with refugees on ART (*n* = 73) and HIV clinic staff (*n* = 4) in Nakivale Refugee Settlement in southwest Uganda from March to July 2011. Three analysts used a conventional content analysis approach to evaluate these data.

**Results:**

Refugees described profound motivation to adhere to ART and employed adherence strategies to facilitate success despite the austere setting. However, refugees spoke of specific hardships living in Nakivale that served as barriers to ART adherence, including difficulty accessing clinic when ill, food insecurity, drug stockouts, and violence and unrest in the settlement. For some refugees, need for ART inextricably linked them to the HIV clinic and prevented them from transitioning permanently away from the settlement.

**Conclusions:**

By learning about refugees’ experiences we can design informed interventions to enhance ART adherence, thus minimizing morbidity and mortality, preventing transmission of HIV, and supporting refugees’ abilities to move freely toward repatriation, resettlement or integration in their host country.

## Background

Adherence to antiretroviral therapy (ART) can help people with HIV live healthier lives and reduces the risk of HIV transmission [1]. However, people living in sub-Saharan Africa face poverty-related barriers (i.e. competing demands, transport costs, food insecurity), institutional barriers (i.e. overburdened healthcare facilities, limited access to mental health care), and political/cultural barriers (i.e. controversies in ART provision, migration, traditional healing beliefs, limited health literacy, gender inequality) [2–5]. The 4.4 million refugees living in sub-Saharan Africa [6] may be uniquely vulnerable to interruptions in ART, given they suffer from disrupted social networks, few livelihood opportunities, threats to their security, increased susceptibility to mental health problems, and difficulties accessing basic needs [7–10]. Understanding and addressing barriers for refugees on ART is important to help tailor interventions for this population to facilitate adherence success.

Initial progress has been made in understanding factors influencing adherence for refugee and internally displaced conflict-affected populations in sub-Saharan Africa. ART can be successfully provided and adhered to in conflict and humanitarian settings [11–15]. However, significant barriers still exist. In a study in Northern Uganda with conflict-affected people on ART, participants described adherence barriers including security while attending clinic, food insecurity, distance to health centers and limited access to health providers [16]. A study comparing experiences of refugees on ART in a camp-based HIV clinic in Kenya and an urban HIV clinic in Malaysia found that in both settings, the main threats to ART adherence were migration and insecurity (food, health system and emotional insecurity) [17]; this is the single study on barriers to ART adherence among a refugee population in sub-Saharan Africa to date.

Our aim was to further investigate experiences of refugees accessing and taking ART while living in a post-conflict settlement in sub-Saharan Africa. Our goal was to understand facilitators and barriers to ART adherence as well as ways to improve adherence. Additionally, we wanted to learn about refugees’ thoughts regarding plans to relocate away from the refugee settlement and if these plans impact their perceptions of ART. To do this, we interviewed refugees on ART and HIV clinic staff in Nakivale Refugee Settlement in southwestern Uganda.

## Methods

### Study design and setting

These data were collected as part of a qualitative study evaluating both refugees’ experiences accessing HIV testing and clinical care, including experiences adhering to ART. Findings related both to HIV testing and social support were reported previously [8, 18]. This paper examines refugees’ experiences and HIV clinic staff perceptions related to access and adherence to ART by refugees attending HIV clinic. This study took place at Nakivale Refugee Settlement, in rural southwest Uganda from March to July 2011. Nakivale was officially recognized as a settlement in 1960 and spans 185 km^2^ [19]. At the time of data collection, there were approximately 56,000 refugees from 9 countries in Nakivale (57% Democratic Republic of the Congo, 22% Rwanda, 13% Somalia, 6% Burundi, 2% Eritrea, Ethiopia, Sudan, Kenya, and Liberia) [20]. This study took place at the HIV clinic at Nakivale Health Center, run by Deutsche Gesellschaft fur Internationale Zusammenarbeit (GIZ), a German governmental organization. At the time of this study, Nakivale Health Center was the primary provider of HIV testing and ART in Nakivale. Distribution of ART in Nakivale began in October of 2008. At the time of this study, based on Uganda national guidelines, those with CD4 count < 250 cells/μL were offered ART irrespective of clinical stage and those with CD4 count between 250 and 350 cells/μL were offered ART if symptomatic (WHO Stage III), co-infected with tuberculosis, or pregnant [21]. While scale-up of ART occurred during the study period, at the start of enrollment only 83 (13%) of the 659 HIV clinic attendees at Nakivale Health Center were on ART. Though data on HIV prevalence in Nakivale during the study period are not available, prevalence rates in the refugees’ countries of origin ranged from 1.1% to 6.3% [22] and a subsequent HIV testing intervention study at Nakivale Health Center revealed a prevalence of 3.3–4.5% [23]. Additionally, ensuing research at this site including refugees and Ugandan nationals that participated in HIV screening demonstrated that HIV prevalence was lower among refugees than Uganda nationals (2% vs 9% respectively) [24] and that 54% (CI: 47–60%) of newly diagnosed HIV-positive individuals link to HIV clinical care in the settlement [25].

### Subject selection

We used a convenience sampling approach to maximize enrollment by offering participation to all eligible individuals as they came to their HIV clinic appointment at the Nakivale Health Center. We recruited participants from the 83 clients on ART at study initiation, as well as those that initiated care after the study began. Inclusion criteria were: 1) being either a refugee receiving ART from the HIV clinic at Nakivale Health Center or HIV clinic staff, 2) ≥18 years of age, and 3) willing and able to give informed consent.

### Ethics approval and consent to participate

This study was approved by the Uganda National Council of Science and Technology (Kampala, Uganda; SS 2408) and the Partners Human Research Committee (Boston, MA, USA; 2010-P-001963/1). Written informed consent was obtained from all study participants in English, Kiswahili or Kinyarwanda. Participation was voluntary and there was no remuneration. Consent forms were read aloud to potential participants. The consent form included statements reminding individuals that this research was distinct from receiving clinical care and they could choose to not participate.

### Data collection

In-person interviews were conducted by a local male refugee research assistant from the Democratic Republic of the Congo fluent in the three interview languages (English, Kiswahili and Kinyarwanda) and trained in qualitative data collection techniques. Interviews took place at the HIV clinic at Nakivale Health Center in a private room. The interviews were semi-structured to cover core topics. Each topic was introduced using a number of open-ended questions to facilitate consistency while allowing for unanticipated material to be introduced by research participants. Additionally, follow-up probes were open-ended or specific to the participant’s response rather than based on a preexisting theory. Core topics included refugee experiences taking ART, stories of missed doses, feelings regarding missed or late medications, factors making ART adherence difficult, factors facilitating ART adherence, and stories of when ART was not available. Additionally, perspectives on how to improve future adherence to ART were explored. Participants were asked if they had plans to relocate from Nakivale, as well as their thoughts on what would happen regarding taking ART during this transition. The other focus of the interviews was experiences accessing HIV testing and clinical care; these data were published previously [8, 18].

After informed consent, interviews were audio-recorded and lasted 50 min on average. Upon interview completion, the research assistant produced a detailed complete transcript of the interview directly into English using the audio recording and written notes. These were verbatim accounts excluding non-essential content such as repeated phrases and hesitations.

### Data analysis

A conventional content analysis approach was used to understand refugees’ experiences accessing and adhering to ART while living in Nakivale. In conventional content analysis, coded categories are derived from the text. This differs from directed content analysis where analysis beings with a theory or related research findings to guide the initial codes, and from summative content analysis which relies on counting and comparing keywords or content prior to interpretation of data [26]. Given the paucity of literature on the topic of refugees and ART adherence, a conventional content analysis approach was selected to obtain information from the study participants without imposing preconceived categories and theoretical perspectives [26]. Three analysts (KNO, SAR, and NRP) individually reviewed all the transcripts and inductively derived codes based on content similarity within the text. Sections of the text were titled, described and informed by interview excerpts. These groupings were revised during an iterative process of reviewing the content and returning to the data. Analysts discussed discrepancies until consensus was reached. The major categories identified were: motivation to adhere to ART, ART adherence strategies, circumstances leading to ART interruption, and fear of leaving ART clinic. A single analyst (KNO) reviewed the grouped content and organized it to form an explanation regarding the impact of ART adherence on refugees’ movement, and to suggest potential means to promote ART adherence in this setting. Frequent returns to the data helped to ensure that the explanation was supported by interview content.

## Results

### Study participants

There were 83 individuals receiving ART from the GIZ clinic at study initiation. With ongoing ART scale-up, by the end of data collection five months later, there were 215 individuals on ART. Seventy-three refugees were invited to participate and all accepted. All 5 HIV clinic staff were offered participation and 4 agreed. The final data set consists of 65 records (61 refugee participants, 4 clinic staff). There are 63 transcripts produced from audio recordings and 2 written transcripts where the interviewee opted to forgo audio recording. Twelve transcripts were lost when an encrypted laptop was stolen. The research staff member that conducted the interviews reported there were no notable differences in the content of the lost transcripts.

### Characteristics of refugee participants (*N* = 61)

Refugee participants were predominantly female (59%, *n* = 36). The average age was 40 years and the average length of time spent living in Nakivale was 9 years. Refugee participants reported an average time spent in formal education of 4.5 years. Refugee participants’ countries of origin included Rwanda (74%, *n* = 45), the Democratic Republic of the Congo (18%, *n* = 11), Burundi (7%, *n* = 4), and Sudan (< 2%, *n* = 1). The majority were Christian (82%, *n* = 50), and the remainder were Muslim (16%, *n* = 10) and Jehovah’s Witness (< 2%, *n* = 1). Most of refugee participants were married (64%, *n* = 39), while the rest were widowed (23%, *n* = 14), divorced (11%, *n* = 7), or single (< 2%, n = 1). Refugees reported an average travel time of 2 h and a cost of 3500 Ugandan Schillings (approximately $1.50 USD) for one-way travel to clinic.

### Characteristics of HIV clinic staff participants (*N* = 4)

Clinic staff participants were half female and had an average age of 27 years. They were all Ugandan and they had an average of 16 years of education. There were three Christians and one Muslim.

### Qualitative results

#### Refugees expressed motivation to adhere to ART

Refugees described a profound motivation to adhere to ART, often because the medications improved their health. One 59-year-old male refugee told, “*There is not even a single day I have been late even for one hour because I know that they [ART] are very important.”* Another 49-year-old male reflected, “*There is not even a single reason why I can miss a dose or forget to take medication on time because I got them after suffering for long.”* Another 38-year-old female refugee valued the medication as any other life-sustaining necessity, “*I decided to love tablets as I love to eat the food”.*

It was evident that participants listened to instructions from clinicians, valued their recommendations, and worked hard to meet their expectations. One female refugee participant recalled, “*Doctors taught us how to take our medication on a daily basis and told us to come when we still have a balance for at least one day. They told us it is very dangerous to miss to take the drugs not even a single dose. So I make sure I don’t miss to take my drugs.”* Most refugees even took their medications on an empty stomach despite side effects if there was no alternative. As one 33-year-old woman stated, *“Sometimes the drugs make us bad because of not having what to eat and drink. But this does not deter us from taking them.”* Some spoke of the negative side-effects brought on by delays in medication doses as a reminder and a motivator to adhere. Another 33-year-old female refugee said, “I forgot to take my drugs for two hours. Then I started to feel some pains in my legs… so I remembered I had not taken my drugs.” Conversely, a 41-year-old male refugee said, “I take my antiretroviral every day but when I forget to take them for one hour I feel no effects”.

#### Refugees employed ART adherence strategies to facilitate success

To ensure successful adherence to ART, refugees in Nakivale devised their own adherence strategies. To avoid missing clinic appointments, two participants talked about coming before they were too physically ill to travel. One 27-year-old man said, “*I have never missed to attend to the clinic. Because when I feel I am becoming sick I immediately come.”* Another 31-year-old female refugee said, “*I have never missed [a clinic appointment]. Even when I’m not feeling very well, I accept to come the day before slowly and I sleep around the clinic so that I get drugs.”*

Refugees also used specific medication reminders. One clinic staff member described, *“Some have used their phone and others use the time for BBC news.”* A 48-year-old female refugee explained her plan to place medications in a visible location to serve as a reminder: *“They told us to keep our drugs where we can easily see. So I keep my drugs in this bag of mine, I always hang it where I see.”* A common strategy was to carry ART at all times to avoid missing doses when away from home. A 38-year-old woman refugee explained: *“You see if you are to go to the garden to dig, you must go very early like at 6 am and yet you may be taking your tablets at like 8 am. So it is better to go with your tablets, dig, and then remember to take your tablets at the usual time.”* Others told of using a similar strategy of carrying ART with them when traveling to visit family, when attending weddings, and when looking for work outside the settlement.

Refugees and HIV clinic staff spoke of the importance of having a contingency plan to help avoid medication interruptions. For example, refugees can register an alternative person to pick up the ART if they are unable to go to clinic themselves. Additionally, a 54-year-old female explained available resources for those physically unwell and unable to travel to clinic: *“When you are very weak, you can call boda boda* [motorcycle] *men to come for you. In serious cases the zone chairman is alerted and he can call for the ambulance to come for you”.*

#### Hardships faced by refugees sometimes led to missed ART

Though most refugees told of their dedication to taking ART, some acknowledged times of extreme hardships when they missed doses. Problems hindering ART adherence included difficulty overcoming the distance to clinic when ill, food insecurity, drug stock-outs and shortages, and violence and unrest in the settlement.

### Distance to clinic when ill

Refugees often live far from health centers (average of two hours for refugee participants in this study) and rarely have financial means to hire a bicycle or motorcycle taxi. Therefore when sick and without energy or strength to walk this distance, some people cannot travel to clinic. This is particularly difficult for refugees who have not disclosed their status or who have not built strong social ties on which to rely in the settlement. One 32-year-old female refugee recounted, *“I was very weak and could not manage to move to the clinic and I could not get someone to take me to the clinic, because I had not revealed that I was HIV positive.”* A 55-year-old Congolese female who said it took her five hours walking to travel to clinic said, “I will never come back to get the drugs from here. I will be getting my drugs from [village], or they should send my drugs to [village], because when I come here [to the clinic], hunger! And going back we reach at home like 9:00 PM and I fail to prepare food and I sleep hungry”.

### Food insecurity

Many spoke of food scarcity as a cause of medication interruption. A 32-year-old female participant explained, “*That [medication interruption] happened only when the [food distributing organization] failed to give us some food in [year] and I could not take the ARVs. I became very weak.”* After some time in the settlement, refugees receive less access to distributed food. One 45-year-old male refugee participant told of the suffering faced when their food ration card was taken away, “*I had my card, but it was cancelled... Now I don’t get food like others. That is why drugs tend to make me weak.”* Additionally, the food eaten in the settlement often requires considerable time to prepare, and on clinic days, many do not have time to prepare food. One 55-year-old female refugee who lived far from clinic recalled, “... *going back we reach at home like at 9 pm and I fail to prepare food and I sleep hungry*”.

### Drug stock-outs and shortages

There were times when medication interruptions occurred because of drug stock-outs. One 30-year-old female refugee who was receiving drugs from Nakivale Health Center via a satellite clinic described a time when the ART was not delivered, stating, “*A whole month was almost ending without I get drugs from here because in our camp they used to tell us they had phoned Nakivale to bring us drugs, but nothing was coming... they refused to bring me [to Nakivale] or bring drugs and I had no transport..*.”. A clinic staff research participant explained that during times of drug shortages, refugees are sometimes given a two-week supply of ART instead of the normal one-month supply.

### Violence and unrest

Some spoke of unrest in the settlement causing absence of healthcare personnel and therefore interruptions in ART distribution. A 37-year-old male refugee told, *“...there was fighting here when the [settlement leader] had conspired to kill a person and the doctors never came to work. That is the only day I never got the drugs.”* In another instance, a refugee was killed in Nakivale causing staff to flee to the nearest city. A 43-year-old male refugee explained, *“There are some days when there was minor insecurity here and one refugee died. It was our day for receiving drugs, but when we came to the clinic, we did not get drugs because all people in charge of the clinic had gone to [nearest city]… we stayed for a whole week without taking drugs.”* During another time of violent conflict in the settlement, refugees physically could not reach clinic. An affected 35-year-old female refugee explained how she had to find medications from an alternate source, “*there was a time when the [ethnic group] had fought and blocked the road and were breaking the car screens that were passing their way... we had to go to [nearest city] to get our drugs, because it was an emergency”.*

#### Missed ART doses sometimes occurred when refugees were away from home

Medication interruptions occurred during travel away from home. Two refugees told of missing ART doses because of traveling for a wedding or a funeral. For some refugees, medication interruptions occurred when traveling for social visits. One 49-year-old male stated, *“Recently, I missed for one day because I had taken a visit to someone and could not carry the drugs with me.”* These periods of travel are not always planned in advance. A 48-year-old female refugee said, “*If I have gone for an unprepared journey, that’s when I can miss [medications] for one day.”* Study participants told of instances when leaving their homes to seek work led to missed doses of ART. One 35-year-old female refugee admitted, *“Sometimes when I go out to look for a living I forget to take the drugs with me, and hence take the drugs late.”* A 36-year-old male told of a specific time when he was working outside the settlement and had a long interruption of ART, recalling, *“There is a time I left the camp to work… and I missed the [ART] for two months”.*

#### Fear of inability to access ART prevented some refugees from permanently leaving the settlement

For a variety of reasons, some refugees fear that moving away from the health clinic in Nakivale could mean losing access to ART. One 50-year-old male refugee believed he would be incarcerated upon return to his home country and thus be unable to access ART. Other refugees described prior experiences trying to leave Nakivale, only to return after facing difficulty enrolling in HIV care. Some refugees felt physically tied to Nakivale because of their health status. A 54-year-old female refugee said, “*The reason why I cannot go from here is that I have no strength to go somewhere else.”* Several noted the importance of access to ART when considering relocating away from the settlement. A 28-year-old woman said, “*If I can get someone to help me to leave this place and I continue to get drugs I can go.”* Another 47-year-old male refugee explained, “*For me my life depends on these drugs. Even if you tell me that you are taking me to America, if there are no drugs there I cannot go”.*

ART adherence was especially difficult for HIV clinic attendees who tried to move outside of Uganda. Medical staff spoke of methods to help refugees transfer their medical care; however, these processes were not always employed. A 48-year-old female refugee who tried to repatriate to her native country before returning to Nakivale recalled, *“When I went back to [country of origin], I did not continue to take the drugs because I had not gone with a transfer [form], so they had to start the whole process of testing. So I had to spend 15 days without taking the drugs.”* Some refugees shared fears of being forced to repatriate quickly without time to plan ahead for their medical needs. One 39-year-old female refugee voiced, “...*people came in the night at 3:00 am to take all refugees who were in [reception center in refugee settlement] or when they came and took [ethnic group] refugees by force. If you forget your drugs you can suffer before you get assistance from the new place.”* One 40-year-old female refugee affected by such an incident who subsequently moved back to Nakivale described, *“In July last year they forcefully repatriated some [ethnic group]. I was among them. I reached [home country] and I had gone without drugs, I had left them at home… Getting drugs from [home country] is not easy! They enroll you and teach you for two weeks before you are given the drugs”.*

## Discussion

In this qualitative study of 73 refugees on ART and 4 HIV clinic staff in Nakivale Refugee Settlement, we sought to learn about refugees’ experiences taking ART while living in a post-conflict refugee settlement in sub-Saharan Africa. Motivation to attend clinic was a predominant theme among refugee study participants, who described innovative strategies to facilitate ART adherence despite the austere setting. However, refugees in the study also spoke of difficulties adhering to ART because of problems overcoming the distance to clinic when ill, food insecurity, drug stock-outs and shortages, and violence and unrest in the settlement. Refugees and HIV clinic staff spoke of missed ART doses when refugees traveled away from the settlement. Concerns that leaving the settlement could disrupt access to ART prevented some refugees from permanently leaving the settlement.

Numerous problems refugees confronted with ART adherence are comparable to those faced by others living in low and middle income countries, especially those barriers relating to poverty. Refugee participants told of difficulties accessing care when they live far from clinic, particularly during times of illness, similar to other sub-Saharan African populations [27, 28]. Additionally, food insecurity was an adherence barrier noted in our study and has been demonstrated to be a problem in other sub-Saharan Africa countries [29]. Drug stock-outs were a reoccurring theme resulting in adherence difficulties for refugees as found elsewhere in sub-Saharan Africa [30]. Refugees also struggle to adhere during times of travel away from the settlement [31].

Some adherence barriers noted by refugees are seemingly more prominent in the refugee context. Violence leading to disruptions in healthcare delivery and medication distribution was notable in our study. While there are war-torn countries such as the Democratic Republic of the Congo where violent conflict disrupts medical care [32], barriers to healthcare because of violence and unrest are not as common for people living in stable settings in sub-Saharan Africa. Also, refugees in our study reported difficulty with adherence when travelling to seek work. The extremely limited livelihood opportunities within the refugee settlement [33] may lead to more frequent travel as refugees seek work.

The themes that emerged from these data on understanding ART adherence among a refugee population, correspond with aspects of the health belief model [34]. The health belief model originated in the 1950s at the United States Public Health Service, as part of an effort to understand the failure of tuberculosis screening programs. This model asserts that individual’s beliefs about health problems, their perceptions regarding benefits and barriers to action, and their self-efficacy, explain their engagement or lack of engagement in health-promoting behaviors [34]. Participants in this study expressed a desire to adhere and developed strategies to facilitate adherence because they believed their health was declining or had the potential to decline, and they perceived ART as beneficial to guarding their health. There were instances when perceived barriers prevented ART adherence, such as when individuals thought they were too ill to travel to clinic or when they were concerned that eating an insufficient quantity of food while taking ART might make them more hungry. As stipulated in the health belief model, these were instances when perceived benefits did not outweigh the perceived barriers to ART adherence. Finally, the notion of self-efficacy, an individual’s perception of his or her ability to perform a behavior, may help explain some differences in ART adherence among our study participants.

The intention of the United Nations High Commissioner for Refugees (UNHCR) is that refugees will ultimately attain a “durable solution” – either repatriation (returning to one’s country of origin), resettlement (permanently moving to an asylum country/third country), or local integration (assimilation into the host country) [33, 35]. However, for many refugees in our study, moving away from the HIV clinic was not perceived as an option. They described difficulties establishing care in a new HIV clinic, feeling too ill to relocate, or becoming over burdened by the time needed to attend clinic to take steps toward achieving a durable solution. While HIV clinic staff spoke of transfer of care processes, refugee participants seemed unaware of the measures in place to help them move away from clinic. The need for refugees living with HIV to access ART may jeopardize their “Access to Durable Solutions,” which is one of the UNHCR’s “10 Key Points on HIV/AIDS and the Protection of Refugees, IDPs and Other Persons of Concern” [36].

While international guidelines call for freedom of movement for those living with HIV [37–40], how to best enable movement for refugees warrants further refugee-focused research. Refugees who are stable on ART could be permitted 3–6 months of ART for planned periods of time away from clinic [11, 41]. Permission from HIV clinicians and the Ugandan Ministry of Health to access HIV-related care from any clinic in Uganda could be given to refugees to help as they travel to seek work around the country. An effort could be made to maintain a uniform medical record with duplicate copies kept by both the HIV clinic and the individual on ART. This could facilitate movement within Uganda as well as transfer of care for those moving away to other countries. While these interventions could improve ART adherence for refugees, it is reasonable to expect that other non-refugee mobile populations (truck drivers or seasonal workers) would benefit as well.

Learning from our qualitative data and drawing from research outside of refugee populations, there are a number of potential avenues to promote ART adherence in the refugee settlement environment. Peer support has led to improvements in low- and middle-income countries [42]. To provide peer support and to help overcome the barrier of distance to clinic, small groups of ART patients could take turns collecting medications at the health facility and distributing among group members [43]. As identified in a study of conflict-affected internally displaced people in Northern Uganda, ways to improve ART adherence in this humanitarian setting might include mobile health teams, increased security, and restocking of drugs with regularity [16]. Pertinent lessons from a Médecins Sans Frontiéres (MSF) HIV program in the Democratic Republic of the Congo included educating on the importance of ART adherence, decentralizing care, establishing treatment information cards and duplicate medical records, and cooperating with HIV treatment facilities in neighboring regions [32].

These data must be interpreted in the context of the study design. The study population included the first people on ART at the health center. These people may have been more motivated than other refugees with HIV to adhere to their medications. This study did not include people who had stopped taking ART or dropped out of clinical care. As such, relevant topics could not be explored, including: reasons people stop taking ART, reasons people drop out of care, and barriers to reengaging in care. Additionally, the study did not include patients who successfully achieved a durable solution, which may affect the results. In Nakivale, refugees live in zones with others from the same country of origin. Country groups living closer to clinic are likely overrepresented in this study; Rwandans made up 74% of the refugee participants. Furthermore, participants living closer to clinic may face different adherence barriers than those living farther from clinic. There was a potential reporting bias as the research assistant’s gender, his country of origin, or the location of the interviews at the health clinic, may have led some participants to be selective with the information they shared. It may also be that participants over-reported their adherence success. Without prior research in this setting on HIV testing and care, the interview guides were intentionally designed to use an inductive approach to identify as many different barriers and facilitators to HIV care as possible, without putting constraints on participant responses by pursuing specific areas of inquiry. As such, potentially relevant barriers including stigma, non-disordered psychological distress, mental disorders (including post-traumatic stress disorder, depressive disorder and substance use disorders), and exposure to gender-based violence were not specifically inquired about and may have been too sensitive for study participants to introduce voluntarily. Finally, this study was conducted in one refugee settlement in sub-Saharan Africa and therefore the findings should be explored in other humanitarian settings to better understand if the results are relevant among other humanitarian-crisis affected populations.

## Conclusions

Despite significant adversity, refugee participants on ART were committed to adhering to medications while living in the refugee settlement. ART adherence for refugees was most vulnerable during times of extreme hardship and during travel away from the HIV clinic. Freedom of movement is essential for refugees seeking a durable solution, and current procedures for ART distribution and HIV clinical care hinder refugees’ abilities to move without restriction. By designing interventions to help refugees with ART adherence, including interventions to facilitate movement while on ART, we can improve the health of refugees, decrease the risk of HIV transmission, and support refugees as they move toward realizing their own durable solution.

## Abbreviation

ART: antiretroviral therapy
